# Distinct Roles for KASH Proteins SINE1 and SINE2 in Guard Cell Actin Reorganization, Calcium Oscillations, and Vacuolar Remodeling

**DOI:** 10.3389/fpls.2022.784342

**Published:** 2022-05-06

**Authors:** Alecia Biel, Morgan Moser, Norman R. Groves, Iris Meier

**Affiliations:** ^1^Department of Molecular Genetics, The Ohio State University, Columbus, OH, United States; ^2^Center for Applied Plant Sciences, The Ohio State University, Columbus, OH, United States; ^3^Center for RNA Biology, The Ohio State University, Columbus, OH, United States

**Keywords:** LINC complex, vacuoles, calcium, actin, cytoskeleton, guard cells

## Abstract

The linker of nucleoskeleton and cytoskeleton (LINC) complex is a protein complex spanning the inner and outer membranes of the nuclear envelope. Outer nuclear membrane KASH proteins interact in the nuclear envelope lumen with inner nuclear membrane SUN proteins. The paralogous Arabidopsis KASH proteins SINE1 and SINE2 function during stomatal dynamics induced by light–dark transitions and ABA. Previous studies have shown F-actin organization, cytoplasmic calcium (Ca^2+^) oscillations, and vacuolar morphology changes are involved in ABA-induced stomatal closure. Here, we show that SINE1 and SINE2 are both required for actin pattern changes during ABA-induced stomatal closure, but influence different, temporally distinguishable steps. External Ca^2+^ partially overrides the mutant defects. ABA-induced cytoplasmic Ca^2+^ oscillations are diminished in *sine2-1* but not *sine1-1*, and this defect can be rescued by both exogenous Ca^2+^ and F-actin depolymerization. We show first evidence for nuclear Ca^2+^ oscillations during ABA-induced stomatal closure, which are disrupted in *sine2-1*. Vacuolar fragmentation is impaired in both mutants and is partially rescued by F-actin depolymerization. Together, these data indicate distinct roles for SINE1 and SINE2 upstream of this network of players involved in ABA-based stomatal closure, suggesting a role for the nuclear surface in guard cell ABA signaling.

## Introduction

The linker of nucleoskeleton and cytoskeleton (LINC) complex serves as a nucleocytoplasmic bridge spanning the nuclear envelope (NE) and connecting to cytoskeletal elements in the cytoplasm and to a network of structural proteins lining the inner nuclear membrane (INM; reviewed in Starr and Fridolfsson, 2010). LINC complexes are composed of Klarsicht/ANC-1/Syne Homology (KASH) outer nuclear membrane proteins and Sad1/UNC-84 (SUN) inner nuclear membrane proteins that interact in the lumen of the NE. Opisthokonts (animals and fungi) and plants have homologous SUN proteins with C-terminal SUN domains located in the NE lumen. However, no proteins with sequence similarity to animal KASH proteins have been discovered in plants (Graumann et al., 2010; Oda and Fukuda, 2011). Recent studies have identified structurally distinct, yet functionally similar, plant KASH proteins with diverse cellular roles (Zhou et al., 2012; Tamura et al., 2013; Graumann et al., 2014; Zhou and Meier, 2014; Zhou et al., 2015; Gumber et al., 2019a,b; Newman-Griffis et al., 2019; Biel et al., 2020a; Moser et al., 2020). The Arabidopsis SINE1 and SINE2 paralogs are plant-specific KASH proteins that are conserved among land plants (Poulet et al., 2016). While SINE1 and SINE2 are both expressed in roots, in leaves SINE1 is expressed only in the stomatal lineage while SINE2 is expressed in trichomes, epidermal and mesophyll cells, and weakly in mature guard cells (Zhou et al., 2014). Both proteins have an N-terminal domain with homology to the armadillo repeat (ARM) domain (Coates, 2003), and the SINE1 ARM domain colocalizes with F-actin (Zhou et al., 2014). We have previously shown that loss of SINE1 or SINE2 increases drought susceptibility, due to a defect in stomatal pore closure, as well as ABA hyposensitivity (Biel et al., 2020a).

ABA-induced stomatal closure involves the activation of guard cell anion channels, increase in cytoplasmic calcium (Ca^2+^), cytoskeleton reorganization, and changes in vacuolar morphology (Eun and Lee, 1997; MacRobbie and Kurup, 2007; Tanaka et al., 2007; Zhao et al., 2011; Jiang et al., 2012; Li et al., 2013, 2014). Actin dynamics are regulated during ABA-induced stomatal closure and are required for stomatal dynamics. F-actin is radially arrayed in open guard cells, depolymerizes at the onset of closure, and is reorganized into a linear, bundled array upon closure (reviewed in Li et al., 2022). Disrupting this reorganization perturbs stomatal dynamics (Kim et al., 1995; Xiao et al., 2004; Jiang et al., 2012; Li et al., 2014; Zhao et al., 2016).

Correlating with changes in actin organization, vacuoles are reorganized during stomatal opening and closing, with small unfused vesicles in the closed state and one large vacuole in the open state (Gao et al., 2005, 2009; MacRobbie and Kurup, 2007; Li et al., 2013). The reorganization of actin filaments likely regulates vacuolar fusion during stomatal opening (Li et al., 2013, 2022). In addition, F-actin was shown to regulate the active state of specific ion channels (MacRobbie and Kurup, 2007; Li et al., 2022).

Ca^2+^ fluctuations in guard cells are linked to changes in the actin cytoskeleton, the regulation of vacuolar structure, and stomatal movements (Xiao et al., 2004; Gao et al., 2005, 2009; MacRobbie and Kurup, 2007; Zhang et al., 2007; Li et al., 2013). Ca^2+^ concentration increases in the cytoplasm of guard cells in response to ABA in an oscillatory mode (McAnish et al., 1990; Allen et al., 1999, 2000; Staxen et al., 1999; Schroeder et al., 2001). This results from Ca^2+^ uptake through plasma membrane Ca^2+^ channels and release from guard cell vacuoles (Ward and Schroeder, 1994; MacRobbie, 2000; Ng et al., 2001; Xiao et al., 2004; MacRobbie and Kurup, 2007; Song et al., 2018). The cytoplasmic Ca^2+^ oscillations are decoded by Ca^2+^ binding proteins such as Ca^2+^-dependent protein kinases and Ca^2+^-sensing receptors and this contributes to stomatal closure (Brandt et al., 2015).

Loss of either SINE1 or SINE2 diminishes stomatal opening in response to light as well as stomatal closing in response to dark or ABA (Biel et al., 2020a). The dynamic range of stomatal apertures during a diurnal cycle is also reduced. External Ca^2+^ can partially rescue the closure defects. After induced stomatal opening, *sine* mutant leaves have an increased drought susceptibility (Biel et al., 2020a). Overexpression of SINE2, but not SINE1, phenocopies the *sine* mutant defects, consistent with the hypothesis that a balance of SINE1 and SINE2 protein abundance in guard cells is important for stomatal closing. Drug-induced actin depolymerization rescues the defect in ABA-induced stomatal closure caused by the loss of SINE1 or SINE2 (Biel et al., 2020a). In addition, JK-induced actin stabilization and polymerization allows *sine1* mutant stomata to close, even in the absence of ABA, but has no effect on *sine2* mutant stomata, suggesting that the two proteins play different roles in conjunction with guard cell actin dynamics (Biel et al., 2020a). The loss of SINE1 and SINE2 also results in compromised microtubule dynamics during ABA-induced stomatal closure (Biel et al., 2020b).

Here, we show that SINE1 and SINE2 regulate different stages of actin re-organization during ABA-induced stomatal closure. Exogenous Ca^2+^ partially overrides the impairments in actin patterning, consistent with the previously reported ability for exogenous Ca^2+^ to rescue the *sine* mutant stomatal closure defect. Cytoplasmic Ca^2+^ oscillations are impaired in *sine2* mutant, but not *sine1* mutant guard cells, and are rescued by the addition of exogenous Ca^2+^ or the disruption of actin organization. Nuclear Ca^2+^ fluctuations occur during ABA-induced stomatal closure and are disrupted in *sine2* mutant guard cells. The loss of SINE1 or SINE2 results in compromised vacuolar fragmentation during ABA-induced stomatal closure, in part due to defects in F-actin changes. Exogenous Ca^2+^ results in stomatal closure without vacuolar fragmentation, regardless of changes in F-actin, in both WT and *sine* mutants. Together, these data support a model proposing that SINE1 and SINE2 both act downstream of ABA, but upstream of changes in actin organization, Ca^2+^, and vacuolar fragmentation, and that the paralogs play clearly distinguishable roles during ABA-induced stomatal closure.

## Materials and Methods

### Plant Material

*Arabidopsis thaliana* (Col-0 ecotype) was grown at 23°C in soil under 16-h light and 8-h dark conditions. For all assays, rosette leaves were collected from 3–4 week-old Arabidopsis plants grown under these conditions. *sine1-1* (SALK_018239C) and *sine2-1* (CS801355), were previously reported, and shown to have no full-length SINE1 or SINE2 mRNA accumulates, respectively (Zhou et al., 2014). 35Spro::GFP-LIFEACT in Col-0 (GFP-LIFEACT; Tolmie et al., 2017) was crossed with *sine1-1* or *sine2-1* and bred until homozygous *sine1-1* and *sine2-1* mutants expressing GFP-LIFEACT were obtained. Genotyping for the *sine1-1* and *sine2-1* insertion alleles were performed as described before (Zhou et al., 2014).

### Cloning

All primers used in cloning and construct generation are outlined in Supplementary Table 1. The guard cell-specific promoter GC1 was cloned from whole seedling genomic DNA (Yang et al., 2008). Restriction sites for enzymes *SacI* and *SpeI* were added to the 5′ and 3′ ends and the amplified fragment was digested with the appropriate restriction enzymes. The GC1 promoter fragment was isolated, purified with the QIAquik PCR Purification kit (Qiagen) and subsequently ligated into a pH2GW7 vector (Takagi et al., 2011).

The Yellow Cameleon 3.6 (YC3.6) Ca^2+^ sensor N-terminally tagged with the rabbit heat stable protein kinase inhibitor α (PKIα) nuclear export signal (NES) was cloned from UBQ10pro::NES-YC3.6 (Krebs et al., 2011). The R-GECO1 sensor, N-terminally tagged with the SV40 nuclear localization signal (NLS) was amplified from CMVpro::NLS-R-GECO1 (Zhao et al., 2011). NES-YC3.6 and NLS-R-GECO1 were cloned into pENTR/D-TOPO vectors (Life Technologies) and then moved to GC1pro::pH2GW7 *via* the Gateway^®^ LR reaction (Life Technologies).

### Generation of Transgenic Plants

Binary vectors containing Ca^2+^ sensors were transformed into either Agrobacterium tumefaciens strains ABI or GV3101 by triparental mating (Wise et al., 2006). The Agrobacterium-mediated floral dip method was used to transform Col-0 ecotype (WT), *sine1-1*, and *sine2-1* backgrounds (Clough and Bent, 1998). Transgenic plants were isolated on Murashige Skoog (MS) plates supplemented with using 30 μg/ml hygromycin, and positive transformants were confirmed by confocal microscopy. One homozygous T2 transgenic line for each background was used for all assays described here.

### Stomatal Aperture Measurements

Stomatal bioassays were performed as previously described (Biel et al., 2020a). Briefly, rosette leaves of 3–4 week-old plants were placed abaxial side up in opening buffer (OB) containing 10 mM MES, 20 μM CaCl_2_, 50 mM KCl, and 1% sucrose at pH 6.15 for 2 h under constant light. Stomatal closing assays were performed immediately after the opening assays, in which leaves were transferred to closing buffer (10 mM MES at pH 6.15) with or without the following treatments, as indicated: 20 μM ABA, 2 mM or 10 mM CaCl_2_ and/or 10 μM LatB (Kang et al., 2002; Jiang et al., 2014). NIS-Elements AR version 3.2 software was used for stomatal aperture measurements.

### Cytoplasmic Calcium Imaging Assay

Arabidopsis expressing the NES-YC3.6 Ca^2+^ sensor driven by the GC1 promoter was imaged using a Nikon Eclipse C90i system. The NES-YC3.6 sensor was excited with 457 nm light, and fluorescence emission was detected between 465 and 505 nm (CFP) and between 530 nm and 570 (cpVenus). Ca^2+^ imaging of stomata was performed as previously described (Behera and Kudla, 2013). Briefly, epidermal peels were mounted on a microscope slide with medical adhesive (Hollister, Libertyville, IL, United States) and incubated in opening buffer (10 mM MES, 20 μM CaCl_2_, 50 mM KCl, and 1% sucrose at pH 6.15) under light for 3 h. A perfusion chamber (Grace Bio-Labs CoverWell) was attached to the slide and the perfusion system was attached to input and output tubing. The fluorescence signal of individual guard cells was observed using confocal microscopy with a 20x oil immersion objective and a 2x digital zoom. To establish a pretreatment baseline, the sample was treated with opening buffer for 5 min and images were captured every 10 s. Next, the epidermal peels were treated with 20 μM ABA (Biel et al., 2020a), 10 mM Ca^2+^ (Jeon et al., 2019) and/or 10 μM LatB (Biel et al., 2020a) and imaged for 45 min. Immediately after the 45-min treatment, guard cells were treated with 100 mM CaCl_2_ for 5 min as a positive control for sensor function. The software NIS-Elements was used to isolate individual guard cells and quantify cytoplasmic or nuclear fluorescence. For NES-YC3.6, the YFP/CFP ratio for each time point was calculated by dividing the mean fluorescence intensity of YFP by the mean fluorescence intensity of CFP. Ca^2+^ peaks were defined as a YFP/CFP ratio increase and decrease of 0.5 A.U. or higher from the baseline.

### Nuclear Calcium Imaging Assay

Arabidopsis expressing the NLS-R-GECO1 Ca^2+^ sensor driven by the GC1 promoter were imaged as described above. The NLS-R-GECO1 sensor was excited with 561 nm light, and its emission was detected between 620 and 650 nm. Stomata were opened using full opening buffer (10 mM MES, 20 μM CaCl_2_, 50 mM KCl, and 1% sucrose at pH 6.15) for 3 h under constant light and epidermal peels were prepared. The epidermal peels were pretreated for 5 min with a modified opening buffer lacking CaCl_2_ (10 mM MES, 50 mM KCl, and 1% sucrose at pH 6.15). This adjustment was required because pretreatment with full opening buffer generated nuclear Ca^2+^ fluctuations prior to ABA addition and thus did not provide an adequate baseline (Supplementary Figure 1A). Removal of the 20 μM CaCl_2_ from the full opening buffer abolished these fluctuations and was thus used to generate the 5-min baseline (Supplementary Figure 1B). The epidermal peels were imaged for 45 min after treatment with 20 μM ABA and then imaged for 5 min after treatment with 100 mM CaCl_2_. The focal plane was adjusted to maintain maximum nuclear fluorescence signal during the time series to minimize any sample drift in the Z direction. RFP fluorescence was normalized by calculating (F − F_0_)/F_0_, where F_0_ represents the average of the baseline values (30 frames) for that experiment. Nuclear Ca^2+^ peaks were determined based on an increase and decrease of 0.5 A.U. or higher from the normalized baseline. Area under the curve (AUC) for each data point was calculated using the following formula: $\left|Y_{1}+Y_{2}\right|/\left(2\times \left(X_{2}+X_{1}\right)\right)$ , where X is time and Y is the normalized Ca^2+^ value. The AUC for each guard cell is the sum of all calculated AUC points for each experiment.

### Confocal Microscopy and Quantification of Filamentous Actin Variations

Confocal microscopy was performed using a Nikon Eclipse C90i system. Images were taken using intact leaves at room temperature with a Plan Flour 60x oil objective (numerical aperture of 1.4, excitation wavelength 488 nm, emission wavelength 516 nm). Z-stacks of 3–18 slices were collected of the cortical layer of guard cells (ensuring exclusion of any nuclear signal) and used for subsequent quantification (Eun et al., 2001; Hwang and Lee, 2001; Gao et al., 2008). The number of filaments, occupancy, and mean angular difference were quantified as described previously with a few modifications (Biel et al., 2020b). For occupancy, a maximum intensity projection was created from the acquired z-stacks, and then the maximum intensity projection of the stoma was separated into individual guard cells. To perform noise reduction, a 1–5 pixel-band-pass filter was applied using the LPX Filter2d plugin (filter = bandpass; bpmode = Gaussian; lo = 1; hi = 5). The image was then binarized by thresholding. For mean angular difference, the binarized image was skeletonized using the LPX Filter2d plugin (filter = filtersbilevelThin; bilevelMode = bilevelThin_). The default settings were used. The average theta for each guard cell was calculated using the LPX Filter2d plugin, using the filter “lineFilters” and the linemode “lineFeature.”

### Vacuolar Morphology Assays

Acridine Orange (AO) staining of vacuoles was performed as described previously with slight modifications (Mathur et al., 2003). Briefly, Arabidopsis leaves with the lower epidermal strip peeled were incubated in 10 μM AO (Sigma-Aldrich Item# A8097) in opening buffer (OB; see above) for 10 to 15 min under dark on a shaker and then washed twice for 2 min with OB. Leaves were then transferred to OB for 60 min under light and then transferred to specified buffers. Confocal microscopy was performed as described above, with the following exceptions: Utilizing an excitation laser of 488 nm, images were captured using 516 nm wavelength and single images were taken mid-cell, where vacuolar morphology was most visible. All images were analyzed using NIS-Elements AR version 3.2 software.

### Statistical Analysis

The number of guard cells analyzed for each line, in all figures, is ≥20. Error bars represent the standard error of means. Letters denote statistical groups (*p* < 0.01), as calculated using one-way ANOVA, followed by a *post hoc* Tukey HSD test. Selected pairwise comparisons were tested using Student’s *t*-test, and asterisks denote statistical significance.

## Results

### ABA-Induced Guard Cell Actin Re-Organization Is Altered in *sine1-1* and *sine2-1*

**Figure 1:**
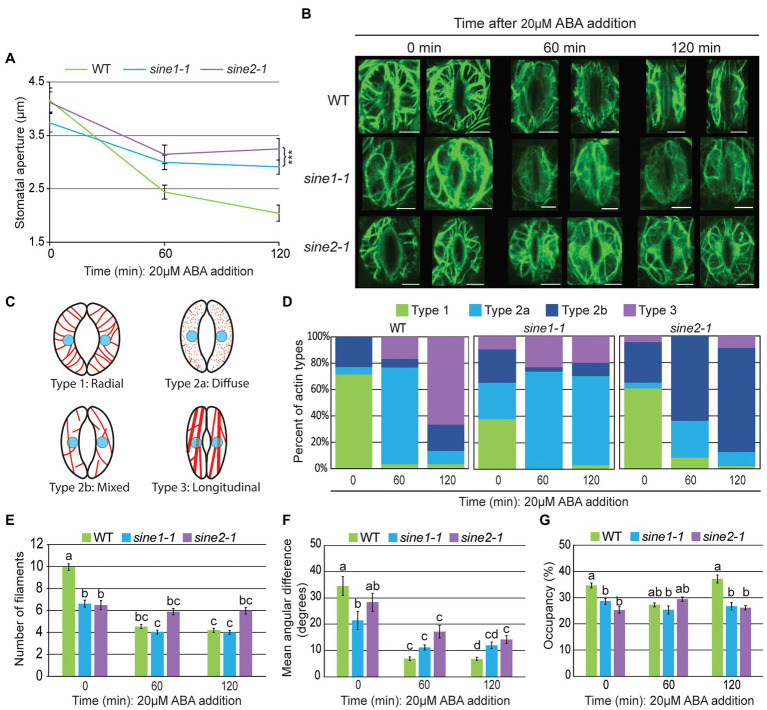
Actin re-organization is disrupted in *sine1-1 and sine2-1* during ABA-induced stomatal closure. **(A)** Stomatal aperture measurements at 0, 60, and 120 min after ABA addition in GFP-LIFEACT transgenic lines. Data are mean values ± SE from three independent experiments. *N* ≥ 30 stomata. Symbols denote statistically significant difference from WT as determined by Student’s *t*-test, with ^***^p < 0.001. **(B)** Representative maximal intensity projections of GFP-LIFEACT in the guard cell cortex at 0, 60, and 120 min after ABA addition. Scale bars = 5 μm. **(C)** Schematic representation of the different actin organization types defined here for quantification in **(D)**. **(D)** Distribution of actin types at 0, 60, and 120 min after ABA addition. *N* ≥ 30 guard cells. For description of types, see text. **(E)** Number of actin filaments. **(F)** The mean angular difference of actin filaments. **(G)** Occupancy (density) of actin filaments. **(E–G)** Data are mean values ± SE from three independent experiments (*N* ≥ 30 guard cells). Statistical significance was calculated using one-way ANOVA followed by a *post-hoc* Tukey HSD test. Lowercase letters denote groups that are statistically different (*p* < 0.01).

The T-DNA insertion lines *sine1-1* and *sine2-1* have been characterized previously and shown to have defects in ABA-induced stomatal closure (Biel et al., 2020a). Col-0 expressing 35S-promoter driven GFP-LIFEACT (hereafter called GFP-LIFEACT; Tolmie et al., 2017) was crossed with *sine1-1* and *sine2-1* to visualize the cortical actin cytoskeleton (see “Materials and Methods”) and F2 lines homozygous for *sine1-1* or *sine2-1* and homozygous or heterozygous for GFP-LIFEACT were used for confocal imaging. To confirm that the actin marker did not influence the previously reported stomatal dynamics (Biel et al., 2020a), stomatal apertures of WT, *sine1-1*, and *sine2-1* GFP-LIFEACT transgenic plants were measured at 0, 60, and 120 min after application of ABA. GFP-LIFEACT-expressing WT closes normally when exposed to ABA while *sine1-1* and *sine2-1* exhibit closing defects in the same range as previously shown for the marker-free lines (Figure 1A; Biel et al., 2020a).

Next, cortical F-actin patterns were imaged at 0, 60, and 120 min after ABA exposure. Two representative images each of maximal intensity projections of the guard cell cortex are shown in Figure 1B. To quantify the actin dynamics during stomatal closure, we used the following actin classification to qualitatively score images, based on Li et al. (2014): Type 1, radially arranged F-actin, with filaments extending from the dorsal to ventral guard cells walls; type 2, diffuse or mixed (radial and longitudinal) filaments; type 3, longitudinally bundled actin cables. We expanded these categories here to account for the loss of filaments seen in *sine1-1* by subclassifying type 2 as type 2a and 2b: type 2a, predominantly diffused signal with fragmented F-actin; and type 2b, predominately mixed filaments with little or no diffuse signal (Figure 1C).

Images were visually scored and sorted into these four groups (Figure 1D). In WT guard cells at 0 min, the most prominent actin filament type was type 1 (radial, 71%), transitioning to type 2a at 60 min (diffuse, 73%) and to type 3 (longitudinal bundles, 67%). In contrast, *sine1-1* guard cells at 0 min had significantly less type 1 (38%), and instead a mix of all three actin types. By 60 min, *sine1-1* guard cells exhibited type 2a (73%) with little change seen at 120 min (type 2a, 67%). Loss of SINE2 resulted in a slight reduction of type 1 at 0 min (61%). At 60 min, *sine2-1* guard cells had transitioned to type 2b (mixed filaments, 64%) with little change in organization seen at 120 min (type 2b, 78%).

Overall, fully open WT guard cells had predominantly radially organized actin filaments, which, upon ABA treatment, underwent reorganization, going through an intermediary, disorganized state (Figure 1D, 60 min), and arriving at a pattern of predominantly thick, longitudinally arrayed bundles (Figure 1D, 120 min). Conversely, *sine* mutants underwent less actin reorganization following exposure to ABA. Loss of SINE1 resulted in a loss of the organized, radial organization of actin filaments at the onset of the assay (Figure 1D, 0 min), which was followed by a state of largely diffuse signal indicative of F-actin depolymerization (Figure 1D, 60 min), that did not recover into an organized pattern by the end of the assay (Figure 1D, 120 min). Loss of SINE2 led to marginally fewer radial filaments prior to ABA perception (Figure 1D, 0 min), but the effect was less pronounced than for the loss of SINE1. F-actin remained mostly intact after ABA treatment, but became less radial and more mixed, and thereafter remained in a mixed state (Figure 1D, 120 min).

To quantify the observed differences in actin organization during ABA-induced stomatal closure, we calculated filament number (Figure 1E), mean angular difference (Figure 1F), and occupancy (Figure 1G). These parameters have been used previously to quantify actin and microtubule (MT) changes in guard cells (Yu et al., 2001, 2020; Higaki et al., 2010; Eisinger et al., 2012a,b; Biel et al., 2020b). Filament number is defined as the number of fluorescent peaks along the mid-width line of the guard cell, as described by Li et al. (2014). The number of filaments in WT guard cells decreases during closure and this change in filament number is used as a proxy to monitor actin bundling (Li et al., 2014). WT guard cells had significantly more filaments than *sine1-1* or *sine2-1* at 0 min (Figure 1E). During ABA-induced stomatal closure, filament number decreased significantly in WT, while *sine1-1* decreased less and was similar to WT, and *sine2-1* showed no significant change.

Mean angular difference was used to quantify F-actin orientation (see section “Materials and Methods”, Higaki et al., 2017; Biel et al., 2020b). Briefly, Z-stacks of individual guard cells were vertically arrayed, isolated, and the cell medial axis was defined. The mean angular difference was measured between actin pixel pairs and the nearest segments of the cell medial axis in the processed images (representative images shown in Supplementary Figure 2; Higaki et al., 2010). An angle closer to 90° represents more transverse filaments, while an angle closer to 0° indicates more longitudinal filaments. In WT, the mean angular difference decreased significantly from 35° at 0 min to 7° at 120 min, consistent with the loss of a radial filament array as reported previously (Higaki et al., 2010; Figure 1F). In *sine1-1* and *sine2-1*, mean angular difference was already significantly lower than WT at 0 min (21^o^ and 28^o^, respectively) and decreased significantly less than WT at 120 min (12^o^ and 14^o^, respectively). This reflects the less pronounced radial arrays at the beginning of the assay and the less pronounced re-organization in both mutants. Thus, consistent with actin type and filament number, this parameter—which reflects changes in actin filament orientation—indicates less change over time in *sine1-1* and *sine2-1*.

Occupancy (displayed in %) measures the average signal distribution within a guard cell and reflects the amount of actin present in filamentous form (Higaki et al., 2010). In WT, occupancy decreased at 60 min, then increased at 120 min, consistent with the visually scored actin reorganization and the literature (Higaki et al., 2010). Consistent with the visual observation, occupancy values for *sine1-1* and *sine2-1* change little over the 120 min time course (Figure 1G).

Together, these results show that loss of SINE1 and SINE2 results in a compromised actin network in open guard cells and overall deficiencies in actin reorganization in response to ABA-induced stomatal closure. Both SINE1 and SINE2 are required for a dense radially arrayed F-actin organization in open guard cells. During guard cell closure, the two mutants affect actin reorganization dynamics at different stages, with *sine2-1* apparently preventing an early depolymerization step (0 to 60 min), while *sine1-1* mutants are arrested in the reorganization of filaments post-depolymerization (60–120 min).

### F-Actin Stabilization Results in Abnormal Actin Patterning in *sine1-1*, Regardless of ABA Presence

**Figure 2:**
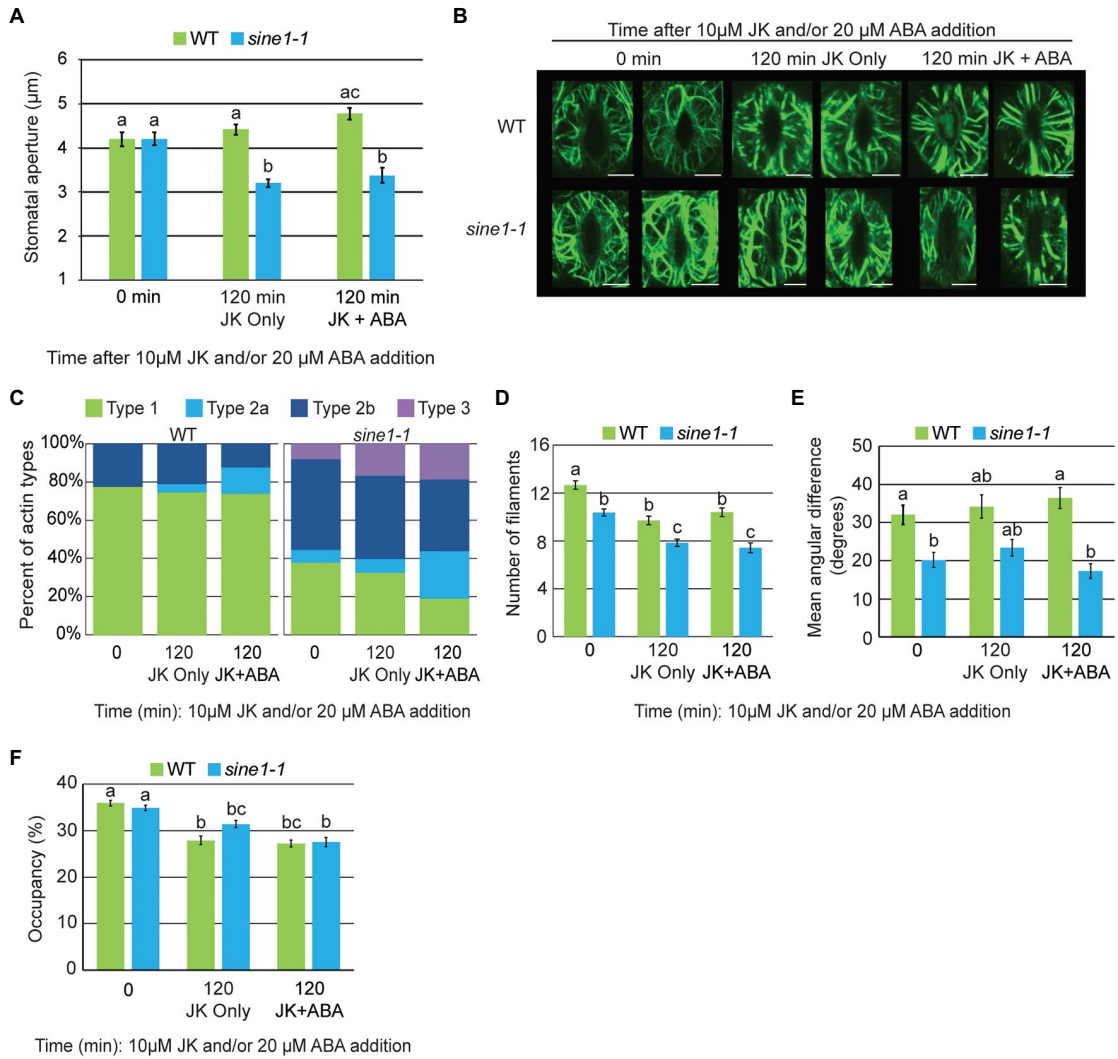
F-actin stabilization results in abnormal actin patterning in *sine1-1* during stomatal closure. **(A)** Stomatal aperture measurements for WT and *sine1-1* GFP-LIFEACT lines at 0 and 120 min after addition of 10 μM JK or 10 μM JK + 20 μM ABA. Data are mean values ± SE from three independent experiments (*N* ≥ 24 stomata). Statistical significance was calculated using Student’s *t*-test, and lowercase letters denote groups that are statistically different (*p* < 0.01). **(B)** Representative maximal intensity projections of actin organization at 0 and 120 min after addition of 10 μM JK or 10 μM JK + 20 μM ABA. Scale bars = 5 μm. **(C)** Distribution of actin types at 0 and 120 min after addition of 10 μM JK or 10 μM JK + 20 μM ABA (*N* ≥ 48 guard cells). **(D)** Actin filament numbers in WT and *sine1-1* guard cells. **(E)** The mean angular difference of actin filaments. **(F)** Occupancy (density) of actin filaments. **(D–F)** Data are mean values ± SE from three independent experiments (*N* ≥ 48 guard cells). Statistical significance was calculated using one-way ANOVA followed by a *post-hoc* Tukey HSD test. Lowercase letters denote groups that are statistically different (*p* < 0.01).

Jasplakinolide (JK) stabilizes and polymerizes F-actin, inhibiting stomatal closure in WT plants (MacRobbie and Kurup, 2007; Li et al., 2014). We have shown previously that the addition of JK, with or without ABA, induces stomatal closure in *sine1-1*, while *sine2-1* mutants remain open, like WT (Biel et al., 2020a). Therefore, we tested here the effect of JK on the actin patterns in WT and *sine1-1* with and without ABA. Because JK had no effect on stomatal closing in *sine2-1*, this line was not tested here. First, we confirmed that the previously reported WT and *sine1-1* closing phenotypes based on JK were not altered by the actin marker. As shown in WT and *sine1-1* without the marker (Biel et al., 2020a), 10 μM JK prevented ABA-based stomatal movement in WT GFP-LIFEACT stomata, while 10 μM JK induced partial closure in *sine1-1* GFP-LIFEACT stomata, with or without concurrent ABA exposure (Figure 2A). This confirms that GFP-LIFEACT does not disturb the JK response in WT or the *sine* mutants.

Next, cortical F-actin patterns were imaged at 0 and 120 min after JK or JK + ABA exposure. At 0 min, the actin filaments are radially arrayed in WT and are in a mixed orientation in *sine1-1*, consistent with Figure 1B (Figure 2B). After 120 min of JK or JK + ABA exposure, F-actin in WT remains radially oriented. In contrast to the loss of F-actin seen after 60 and 120 min of ABA (Figure 1B), *sine1-1* after 120 min of JK or JK + ABA exposure adopts a mixed F-actin pattern, with both longitudinal and radial filaments seen, with some fragmented actin also seen with JK + ABA (Figure 2B). Categorizing actin patterning, as described for Figure 1C, supports this observation (Figure 2C). This suggests that JK prevents the reorganization of actin filaments induced by ABA in WT, likely leading to the suppression of guard cell closure. In *sine1-1* however, JK counteracts the loss of filaments after ABA treatment and inability to re-create an F-actin network. Under these conditions, *sine1-1* stomata can close.

Filament number in WT guard cells decreased slightly with exposure to JK and JK + ABA, but this effect was much reduced compared to ABA treatment (Figures 1E, 2D). Filament number also decreased slightly in *sine1-1* with exposure to JK and JK + ABA (Figure 2D). The mean angular difference is higher in WT than in *sine1-1* at time point 0, and this difference is maintained with JK and JK + ABA treatment, consistent with the higher proportion of type 1 actin in WT stomata (Figures 2C,E). Occupancy at time point 0 was similar between WT and *sine1-1* and decreased slightly by both treatments (Figure 2F). Together, these data indicate that the stabilization of actin filaments by JK overrides the closing defect caused by *sine1-1*, suggesting that the prolonged depolymerized state in the *sine1-1* mutant has an inhibitory effect.

### Calcium Partially Restores Stomatal Closing-Associated Actin Remodeling in *sine1-1* and *sine2-1*

It has been established that ABA signaling results in cytoplasmic Ca^2+^ increases to induce stomatal closure, and that Ca^2+^ alone can stimulate closure (Allen et al., 1999; Hamilton et al., 2000; Kim et al., 2010; An et al., 2016). We have previously shown that exogenous Ca^2+^ partially rescues the *sine1-1* and *sine2-1* defect in ABA-induced stomatal closure, suggesting that SINE1 and SINE2 act downstream of ABA, but upstream of a Ca^2+^-dependent step in the pathway (Biel et al., 2020a). Given that both SINE1 and SINE2 are required for WT-like reorganization of the actin cytoskeleton during stomatal closure, we tested if exogenous Ca^2+^ also partially rescues this defect.

**Figure 3:**
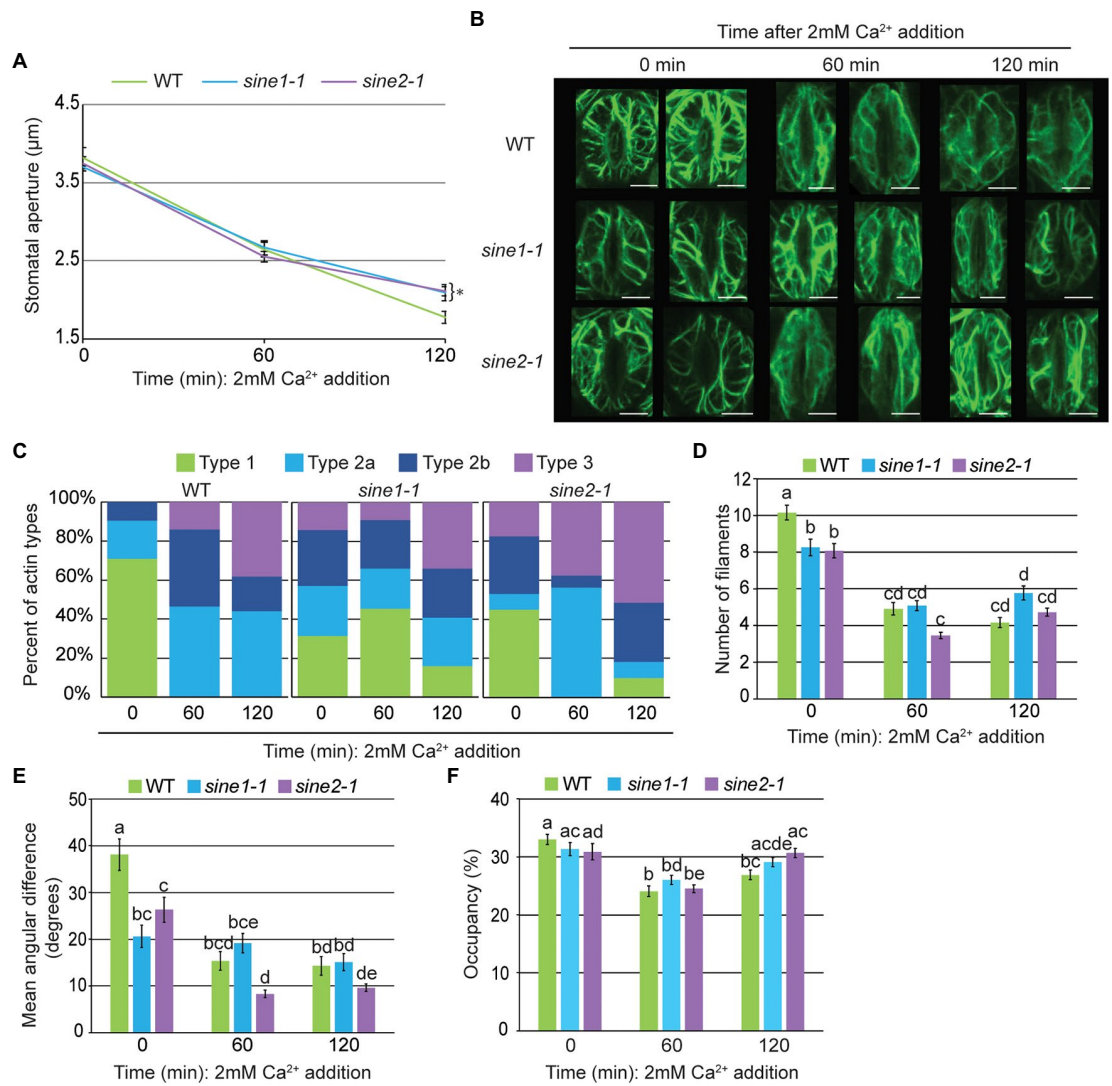
Calcium-induced stomatal closure partially restores actin organization defects observed in *sine* mutants. **(A)** Stomatal aperture measurements in GFP-LIFEACT transgenic lines at 0, 60, and 120 min after addition of 2 mM Ca^2+^. Data are mean values ± SE from three independent experiments (*N* ≥ 24 stomata). Symbols denote statistically significant difference to WT as determined by Student’s *t*-test, with ^*^p < 0.05. **(B)** Representative maximal intensity projections of actin organization at 0, 60, and 120 min after addition of 2 mM Ca^2+^. Scale bars = 5 μm. **(C)** Distribution of actin types at 0, 60, and 120 min after ABA addition. **(D)** Actin filament numbers in WT, *sine1-1*, and *sine2-1* guard cells. **(E)** The mean angular difference of actin filaments. **(F)** Occupancy (density) of actin filaments in WT, *sine1-1*, and *sine2-1*. **(D–F)** Data are mean values ± SE from three independent experiments (*N* ≥ 44 guard cells). Statistical significance was calculated using one-way ANOVA followed by a *post hoc* Tukey HSD test. Lowercase letters denote groups that are statistically different (*p* < 0.01).

Exogenous Ca^2+^ (2 mM) was sufficient to induce stomatal closure in WT GFP-LIFEACT, *sine1-1* GFP-LIFEACT and *sine2-1* GFP-LIFEACT, again indicating that the actin marker does not interfere with Ca^2+^-induced stomatal dynamics (Figure 3A). As seen in the maximal intensity projections in Figure 3B and the visual scoring of actin patterns in Figure 3C, exogenous Ca^2+^ drastically reduced the amount of radial F-actin in WT guard cells (similar to ABA treatment, compare to Figures 1B,D). After 120 min, the signature longitudinal actin bundle pattern appeared in about half of the scored guard cells, suggesting that Ca^2+^-induced actin reorganization was less complete, or slower, than that induced by ABA. Exogenous Ca^2+^ increased the formation of longitudinal filaments in both *sine1-1* and *sine2-1*, which was not seen with ABA. Curiously, *sine1-1* displayed a substantial amount of radial filaments at 60 min after Ca^2+^ addition whereas ABA exposure resulted in a largely diffuse actin pattern (Figure 3C, middle panel vs. Figure 1D, middle panel). While the significance of this stage is not known, 2 mM Ca^2+^ clearly did not cause the accumulation of Type 2a actin observed in *sine1-1* after treatment with ABA. Similarly, Ca^2+^ prevented for *sine2-1* the “arrest” of filament reorganization at a disorganized stage (Type 2b) seen after ABA treatment.

Applying the same quantitative parameters as above, guard cell filament number declined more in the *sine* mutants after exposure to 2 mM Ca^2+^ than after ABA exposure (Figures 3D vs. 1E). No significant change in mean angular difference was revealed after Ca^2+^ exposure in *sine1-1*, consistent with the overall mixed patterns of actin filaments observed (Figures 3C,E). Mean angular difference in *sine2-1* decreased further at 60 min and 120 min following Ca^2+^ treatment, consistent with the overall increase of the type 3 filament pattern (Figures 3C,E). Together, exogenous Ca^2+^ treatment resulted in a similar, if somewhat reduced (or possibly delayed) reorganization of actin in WT, compared to ABA treatment (Figures 3C–F). Both *sine1-1* and *sine2-1* went through a larger degree of actin reorganization than after ABA treatment, resulting in a larger number of guard cells with type 3 pattern at the end of the assay, consistent with the partial induction of stomatal closing in the mutants.

### Loss of SINE2, but Not SINE1, Disrupts Cytoplasmic Calcium Oscillations

**Figure 4:**
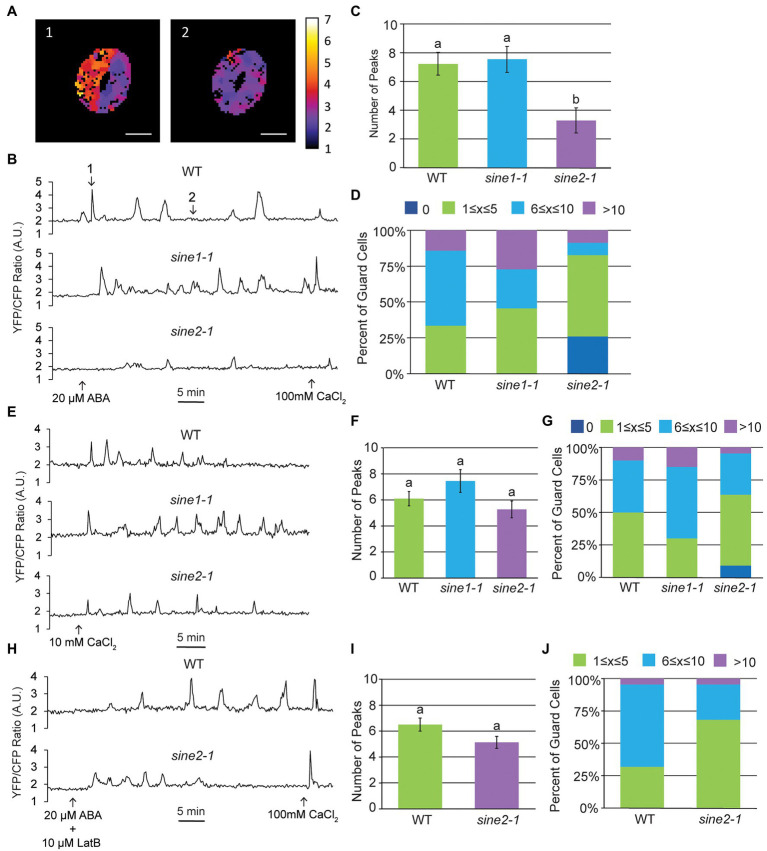
Cytoplasmic calcium oscillation is disrupted in *sine2-1*. **(A)** Ratiometric images of cytoplasmic Ca^2+^ in WT after addition of 20 μM ABA. Images shown are of the guard cell cortex at two individual time points. Scale bars = 10 μm. **(B)** Characteristic WT, *sine1-1*, and *sine2-1* Ca^2+^ oscillations in response to 20 μM ABA, presented as FRET ratio of YFP to CFP fluorescence intensity. Numbers in the WT graph correspond to the image numbers in **(A)**. **(C)** Number of peaks observed after addition of ABA in WT, *sine1-1*, and *sine2-1* guard cells. Statistical significance was calculated using one-way ANOVA followed by a *post-hoc* Tukey HSD test. Lowercase letters denote groups that are statistically different (*p* < 0.01). **(D)** Number of peaks observed were grouped into several categories for WT, *sine1-1*, and *sine2-1*. **(E)** Representative Ca^2+^ oscillations in WT, *sine1-1*, and *sine2-1* in response to exogenous 10 mM CaCl_2_. **(F)** Number of peaks observed after addition of ABA in WT, *sine1-1*, and *sine2-1* guard cells. Statistical significance was calculated using one-way ANOVA followed by a *post-hoc* Tukey HSD test. Lowercase letters denote groups that are statistically different (*p* < 0.01). **(G)** Number of peaks observed were grouped into several categories for WT, *sine1-1*, and *sine2-1*. **(H)** Representative Ca^2+^ oscillations in WT and *sine2-1* after treatment with 20 μM ABA +10 μM LatB. **(I)** Number of peaks observed after addition of 10 μM LatB and 20 μM ABA in WT and *sine2-1* guard cells. Statistical significance was calculated using one-way ANOVA followed by a *post-hoc* Tukey HSD test. Lowercase letters denote groups that are statistically different (*p* < 0.01). **(J)** Number of peaks observed were grouped into several categories for WT and *sine2-1*.

ABA-induced stomatal closure results in oscillatory changes in cytoplasmic Ca^2+^ (McAnish et al., 1990; Allen et al., 1999, 2000; Staxen et al., 1999; Schroeder et al., 2001). Given that our previous data suggest that SINE1 and SINE2 act upstream of a Ca^2+^-dependent step in the ABA pathway, we tested if ABA-induced cytoplasmic Ca^2+^ oscillations are disrupted in *sine* mutants. Using the genetically encoded ratiometric Ca^2+^ sensor YC3.6 tagged with a nuclear export signal (NES-YC3.6), we observed ABA-induced cytoplasmic Ca^2+^ oscillations in WT, *sine1-1*, and *sine2-1* (Figures 4A,B). Counting and averaging the number of Ca^2+^ peaks for 45 min post-ABA addition indicated no significant difference between WT and *sine1-1*, but a significant reduction in peak number for *sine2-1* (Figures 4B,C). The distribution of peak number for WT and *sine* mutants showed an absence of cytoplasmic Ca^2+^ peaks after ABA addition in approximately 25% of *sine2-1* guard cells, but not in either WT or *sine1-1* (Figure 4D). We next tested whether addition of exogenous Ca^2+^ could override the dampening of the cytoplasmic Ca^2+^ response to ABA in *sine2-1* (Figures 4E–G). Both 1 mM and 10 mM external CaCl_2_ have been shown previously to induce comparable cytoplasmic Ca^2+^ oscillations (Allen et al., 2000; Jeon et al., 2019). Here, inducing stomatal closure with 10 mM CaCl_2_ resulted in an average of 6 Ca^2+^ peaks in 45 min in *sine2-1*, similar to WT and *sine1-1* (Figure 4F). The portion of *sine2-1* guard cells with no observable Ca^2+^ oscillations was reduced to 10% (Figure 4G). This suggests that, consistent with the closing phenotype, exogenous Ca^2+^, but not ABA, can trigger cytoplasmic Ca^2+^ fluctuations in *sine2-1*.

We have previously shown that addition of the actin depolymerizing drug Latrunculin B (LatB), in conjunction with ABA, is able to induce closure in *sine* mutants (Biel et al., 2020a). Thus, we tested if disrupting actin organization could also induce WT-like cytoplasmic Ca^2+^ oscillations in *sine2-1* (Figures 4H–J). As a control, GFP-LIFEACT plants were imaged before and after 10 μM LatB treatment in both whole leaves and epidermal peels (Supplementary Figure 3A). Before addition of LatB, actin filaments (F-actin) are seen in both guard cells and the surrounding pavement cells in both whole leaves and epidermal peels. After 60 min LatB treatment in whole leaves, the F-actin network appears more diffuse and by 120 min appears completely disrupted (Supplementary Figure 3A). In epidermal peels, the F-actin network begins to noticeably disintegrate 10 min after LatB treatment (Supplementary Figure 3B). Addition of LatB and ABA induced, on average, 5 peaks per 45 min for *sine2-1*, similar to WT (Figure 4I). No traces without any Ca^2+^ oscillations were observed (Figure 4J). Taken together, these data demonstrate that ABA-induced cytoplasmic Ca^2+^ fluctuations are dampened in *sine2-1* and that both the addition of exogenous Ca^2+^ and the addition of LatB can restore them to near-WT levels. Thus, induced actin depolymerization can override the cytoplasmic Ca^2+^ oscillation defect, consistent with the depolymerization defect of *sine2-1* shown in Figure 1.

### ABA Induces Nuclear Calcium Fluctuations in Guard Cells, Which Are Disrupted in *sine2-1*

Nuclear Ca^2+^ fluctuations have been reported during biotic and abiotic stress as well as root development in a variety of plant species (Capoen et al., 2011; Liang et al., 2014; Charpentier et al., 2016; Charpentier, 2018; Leitão et al., 2019). Here, we tested whether guard cells also undergo nuclear Ca^2+^ fluctuations by using the genetically encoded Ca^2+^ sensor R-GECO1 tagged with a nuclear localization signal (NLS-R-GECO1). When the samples were treated with opening buffer for 5 min to establish a baseline prior to ABA addition, nuclear Ca^2+^ oscillations were observed (Supplementary Figure 1). To determine if this was due to the 20 μM CaCl_2_ in the opening buffer, we compared guard cells exposed for 45 min to the full opening buffer (10 mM MES, 1% sucrose, 50 mM KCl, 20 μM CaCl_2_) or a modified opening buffer lacking Ca^2+^ (10 mM MES, 1% sucrose, 50 mM KCl; Supplementary Figures 1A,B). Nuclear Ca^2+^ fluctuations were observed in full opening buffer but were absent in modified opening buffer, suggesting that 20 μM exogenous CaCl_2_ is sufficient to trigger nuclear Ca^2+^ fluctuations in guard cells. We also tested whether *sine* mutants exposed to full opening buffer exhibit nuclear Ca^2+^ fluctuations (Supplementary Figures 1C,D). Using peak number and peak categorization to compare we observed similar average peak numbers and trends between WT and *sine* mutants (Supplementary Figures 1C,D). Therefore, to establish a baseline needed for ABA-induced Ca^2+^ experiments, we used the modified opening buffer for 5 min prior to ABA addition.

**Figure 5:**
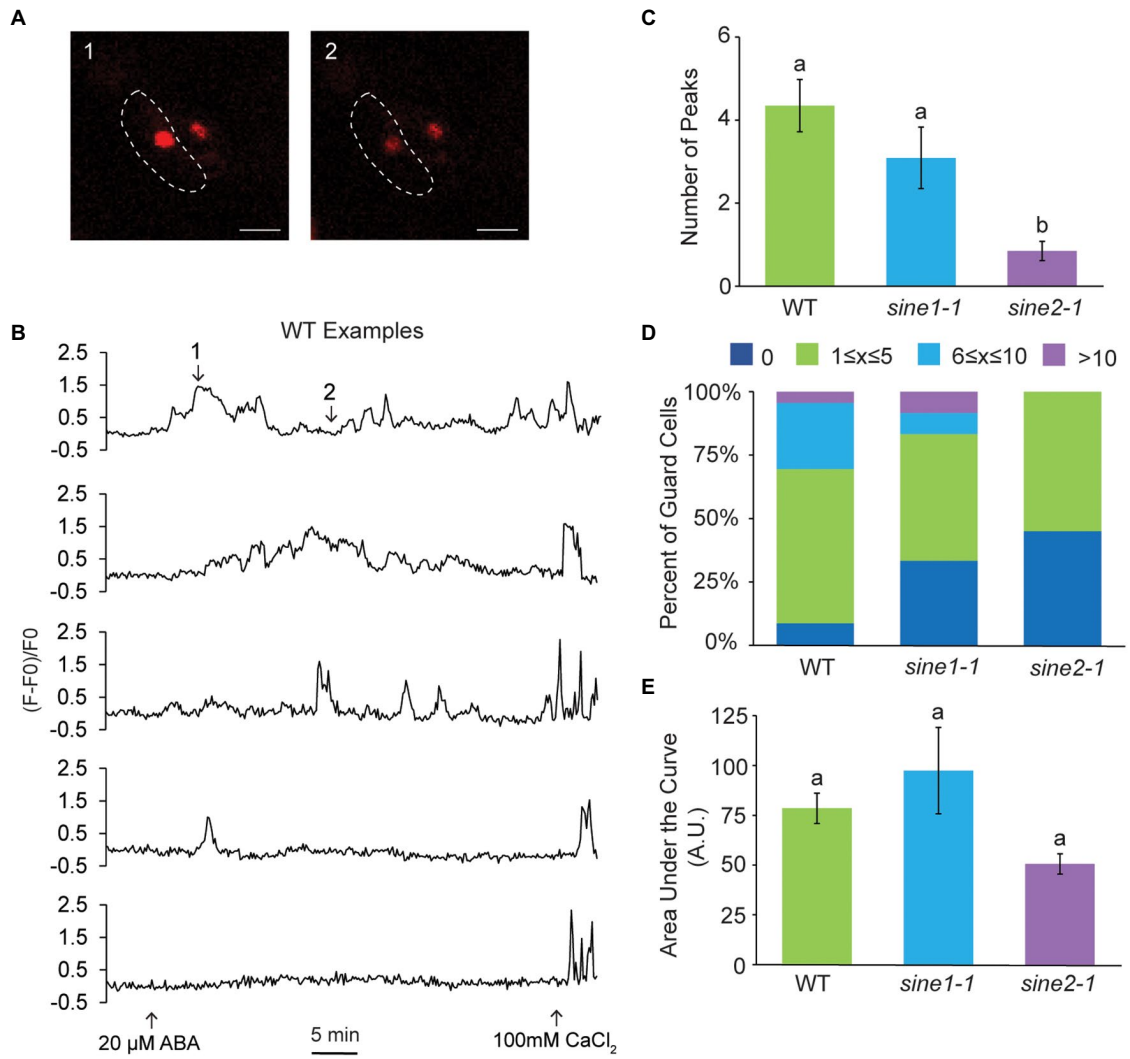
Nuclear calcium fluctuations are disrupted in *sine2-1*. **(A)** Representative images of nuclear Ca^2+^ fluctuations in WT after addition of 20 μM ABA. Images shown are of the guard cell nuclei at two individual time points. The border of the left guard cell is outlined with the dotted line. Scale bars = 10 μm. **(B)** Five examples of nuclear Ca^2+^ fluctuations in WT in response to 20 μM ABA, presented as normalized fluorescence intensity over time. Numbers in the first graph correspond to the image numbers in **(A)**. **(C)** Number of peaks observed after addition of ABA in WT, *sine1-1*, and *sine2-1* guard cells. Statistical significance was calculated using one-way ANOVA followed by a *post-hoc* Tukey HSD test. Lowercase letters denote groups that are statistically different (*p* < 0.01). **(D)** Number of peaks observed were grouped into several categories for WT, *sine1-1*, and *sine2-1*. **(E)** Area under the curve of nuclear Ca^2+^ fluctuations in WT, *sine1-1*, and *sine2-1* after treatment with 20 μM ABA. Statistical significance was calculated using one-way ANOVA followed by a *post-hoc* Tukey HSD test. Lowercase letters denote groups that are statistically different ( *p* < 0.01).

Changes in nuclear Ca^2+^ fluctuations were observed after addition of ABA but appeared irregular and variable compared to the oscillatory nature of cytoplasmic Ca^2+^ (Figures 5A,B). Compared to WT, the average number of peaks in 45 min post ABA addition was significantly reduced in *sine2-1,* but not *sine1-1*, mirroring the results for the cytoplasmic Ca^2+^ oscillations (Figure 5C). This was also reflected in the distribution of peaks per trace (Figure 5D). Given the irregular nature of the nuclear Ca^2+^ fluctuations, we also calculated the area under the curve for WT and *sine* mutants, with qualitatively similar results (see section “Materials and Methods”; Figure 5E). Taken together, these data show evidence for SINE2-influenced nuclear Ca^2+^ fluctuations in guard cells during ABA-induced stomatal closure.

### Loss of SINE1 and SINE2 Compromises Vacuolar Fragmentation During ABA-Induced Stomatal Closure

**Figure 6:**
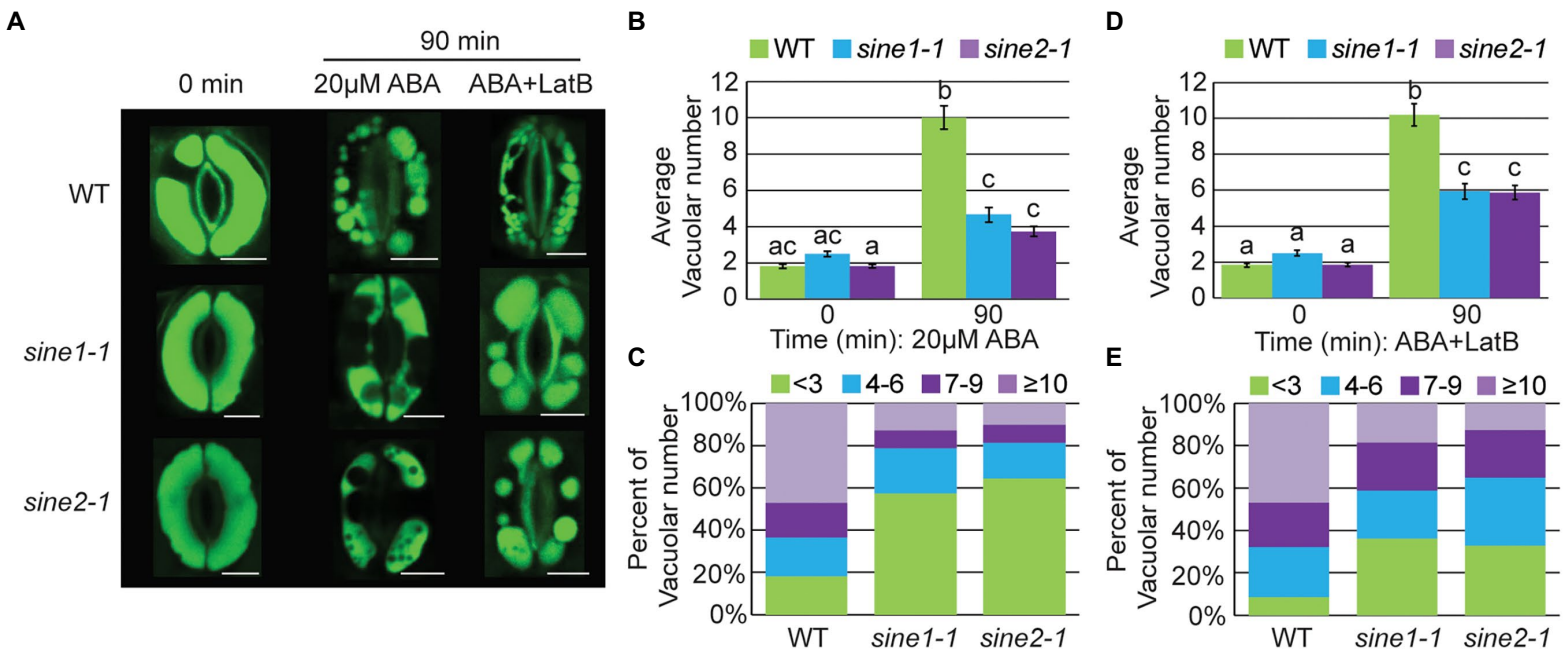
Vacuolar fragmentation is compromised during ABA-induced stomatal closure in *sine* mutants owing, in part, to disruptions in actin organization. **(A)** Representative images of vacuolar morphology at 0 and 90 min after addition of either 20 μM ABA or 20 μM ABA +10 μM LatB. Scale bars = 5 μm. **(B,D)** Average vacuolar number seen in guard cells before and after treatment with **(B)** 20 μM ABA or **(D)** 20 μM ABA +10 μM LatB. Statistical significance was calculated using one-way ANOVA followed by a *post hoc* Tukey HSD test. Lowercase letters denote groups that are statistically different (*p* < 0.01). Data are mean values ± SE from three independent experiments. The number of stomata analyzed for each line is ≥68 guard cells. **(C,E)** Distribution of distinct vacuolar structures observed in guard cells after 90 min of treatment with either **(C)** 20 μM ABA or **(E)** 20 μM ABA +10 μM LatB.

Stomata open and close with concomitant changes in vacuolar morphology and actin reorganization (Fricker and White, 1990; Higaki et al., 2007; MacRobbie and Kurup, 2007; Li et al., 2013; Zheng et al., 2014, 2019). Upon ABA-induced stomatal closure, fused vacuoles of open guard cells undergo fission or constriction (hereafter called fragmentation; Zheng et al., 2014; Song et al., 2018). We tested if SINE1 and SINE2 are involved in vacuolar fragmentation during ABA-induced stomatal closure and if this process depends on actin reorganization. Acridine orange (AO; Fricker and White, 1990; Gao et al., 2005; Higaki et al., 2007; MacRobbie and Kurup, 2007; Li et al., 2013; Zheng et al., 2014) was used to monitor changes in vacuolar morphology during stomatal closure. We defined vacuolar fragmentation by the number of separated AO-containing structures. At 0 min of ABA treatment WT, *sine1-1*, and *sine2-1* exhibited large, fused vacuoles (Figure 6A). After 90 min of ABA exposure, vacuolar fragmentation was seen in WT (Figures 6A–C). Addition of LatB + ABA resulted in similar vacuolar fragmentation as ABA treatment alone (Figures 6A,D,E). Conversely, vacuolar fragmentation after ABA treatment was significantly reduced in *sine1-1* and *sine2-1* (Figures 6A–C). Addition of LatB to ABA treatment increased the number of fragmented vacuoles in *sine1-1 and sine2-1* (Figures 6A,D,E), more so than ABA treatment alone (Supplementary Table 2, first row).

These data suggest that ABA-induced vacuolar fragmentation is compromised in *sine1-1* and *sine2-1*. Depolymerizing F-actin partially overrides this defect, consistent with the rescue of ABA-induced stomatal closure by LatB (Biel et al., 2020a). This is consistent with the assumption that an aspect of the dynamic actin reorganization is required for vacuolar fragmentation, that this is disrupted in the mutants, and that the inhibitory effect caused by this disruption is released by F-actin depolymerization.

### Vacuolar Fragmentation Does Not Occur During Stomatal Closure Induced by Exogenous Calcium, Regardless of Changes to Actin Organization

**Figure 7:**
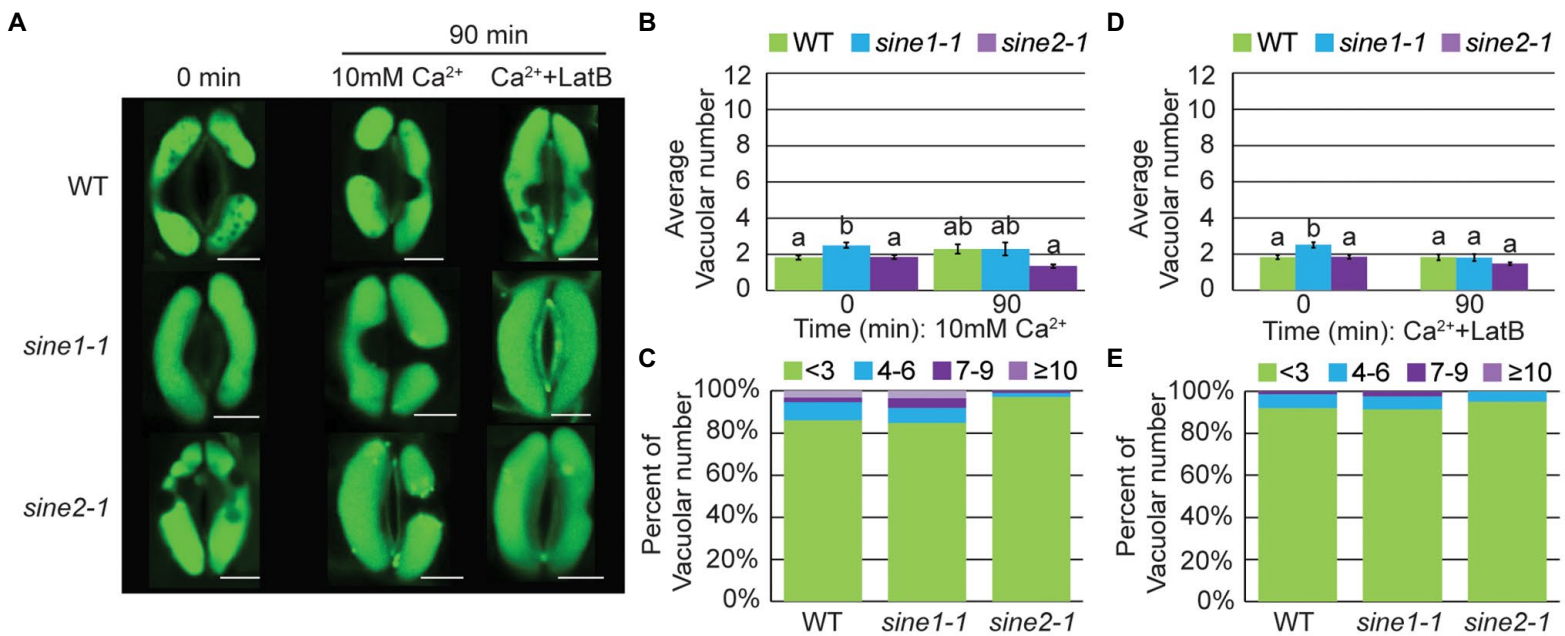
Vacuolar fragmentation does not occur during Ca^2+^-induced stomatal closure, regardless of changes to actin organization. **(A)** Representative images of vacuolar morphology at 0 and 90 min after addition of either 10 mM Ca^2+^ or 10 mM Ca^2+^ + 10 μM LatB. Scale bars = 5 μm. **(B,D)** Average vacuolar number seen in guard cells before and after treatment with (B) 10 mM Ca^2+^ or **(D)** 10 mM Ca^2+^ + 10 μM LatB. Statistical significance was calculated using one-way ANOVA followed by a *post hoc* Tukey HSD test. Lowercase letters denote groups that are statistically different ( *p* < 0.01). Data are mean values ± SE from three independent experiments. The number of stomata analyzed for each line is ≥68 guard cells. **(C,E)** Distribution of distinct vacuolar structures observed in guard cells after 90 min of treatment with either **(C)** 10 mM Ca^2+^ or; **(E)** 10 mM Ca^2+^ + 10 μM LatB.

During stomatal closure and vacuolar fragmentation, there is a rise in the cytoplasmic Ca^2+^ concentration *via* Ca^2+^ influx and Ca^2+^ release from intracellular stores (Ward and Schroeder, 1994; MacRobbie, 2000; Ng et al., 2001; Xiao et al., 2004; MacRobbie and Kurup, 2007; Song et al., 2018). Therefore, we tested whether exogenous Ca^2+^ induces vacuolar fragmentation and if any response is compromised by the loss of SINE1 or SINE2. Stomatal closure assays were performed by applying 10 mM Ca^2+^ and monitoring vacuolar morphology using AO (Figure 7). As shown in Figure 6, WT, *sine1-1*, and *sine2-1* pre-opened stomata at 0 min had large, fused vacuoles (Figure 7A). After 90 min of exposure to 10 mM Ca^2+^, vacuoles of WT, *sine1-1*, and *sine2-1* guard cells were still predominantly large and fused, with little change (Figures 7A–C). Addition of LatB caused no change, WT, *sine1-1*, and *sine2-1* guard cells still exhibited large, fused vacuoles (Figures 7A,D,E). Stomatal closure was normal for both 10 mM Ca^2+^ and 10 mM Ca^2+^ + LatB treated samples (Supplementary Figure 4; Supplementary Table 2). It was noted that exogenous Ca^2+^ in a lower concentration (2 mM) led to a small degree of vacuolar fragmentation in WT and *sine2-1*, significantly less so than ABA but significantly more than 10 mM Ca^2+^ (Supplementary Table 2; Supplementary Figure 5). Taken together, stomatal closure can occur in the absence of vacuolar fragmentation when induced by exogenous Ca^2+^ and, under these conditions, actin depolymerization does not impact vacuolar fragmentation.

## Discussion

Our original hypothesis that SINE1, but not SINE2, has a guard-cell-related function (Zhou et al., 2014; Biel et al., 2020a) was based on three observations. These were: (1) that in leaves SINE1 is specifically expressed in the guard cell lineage, (2) that in *sine1-1*, but not in *sine2-1*, the typically paired guard cell nuclei are positioned more randomly, and (3) that only the SINE1 ARM domain co-localizes in guard cells with F-actin. However, when probing into the guard cell physiology of *sine1-1* and *sine2-1* loss-of function mutants we found that both affected stomatal dynamics, and in many cases in the same ways. However, some differences were also observed, importantly a different interaction of the two mutants with actin-modulating drugs (Biel et al., 2020a).

Thus, one of the key questions was to understand the similarities and differences in the roles of SINE1 and SINE2 in guard cell function. To address this question, we have applied a battery of assays to probe into known cell-biological players of stomatal dynamics. We have focused this analysis on ABA-induced stomatal closure, because of the preponderance of available information. Specifically, we have tested (1) actin re-organization, (2) cytoplasmic and nuclear Ca^2+^ fluctuations, and (3) vacuolar reorganization. In addition, we have investigated the interdependence of these well-characterized cell-biological readouts of stomatal closure and into how loss of SINE1 and SINE2, respectively, affect them.

Numerous studies support the role of ABA in inducing actin organizational changes to mediate stomatal closure and show that the regulation of stomatal movement requires both F-actin polymerization and depolymerization (reviewed in Kim et al., 1995; Hwang et al., 1997; Eun et al., 2001; Lemichez et al., 2001; MacRobbie and Kurup, 2007; Gao et al., 2008, 2009; Higaki et al., 2010; Zhao et al., 2011; Jiang et al., 2012; Li et al., 2014, 2022). Here, we found that while actin remodeling is disrupted in both *sine1-1* and *sine2-1* mutants, the defects occur at different stages of actin remodeling, suggesting that SINE1 and SINE2 play distinct roles during different phases of the closure process. Loss of SINE1 impaired the ability of actin to re-form filaments during reorganization and/or maintain a polymerized state. We have shown previously that JK induced stomatal closure in the absence of SINE1, even without ABA (Biel et al., 2020a). In *sine1-1* guard cells, JK overrides the disappearance of F-actin, thus indicating that a problem with polymerization and/or stabilization of F-actin in *sine1-1* is inhibitory to stomatal closing. We note that the *sine1-1* F-actin organization after JK is distinct from that of WT, with a significant reduction in radial patterning. Thus, rescue of the *sine1-1* closing defect by JK implies that a certain degree of F-actin, but not necessarily an exact organization, is required for the completion of the closing process.

**Figure 8:**
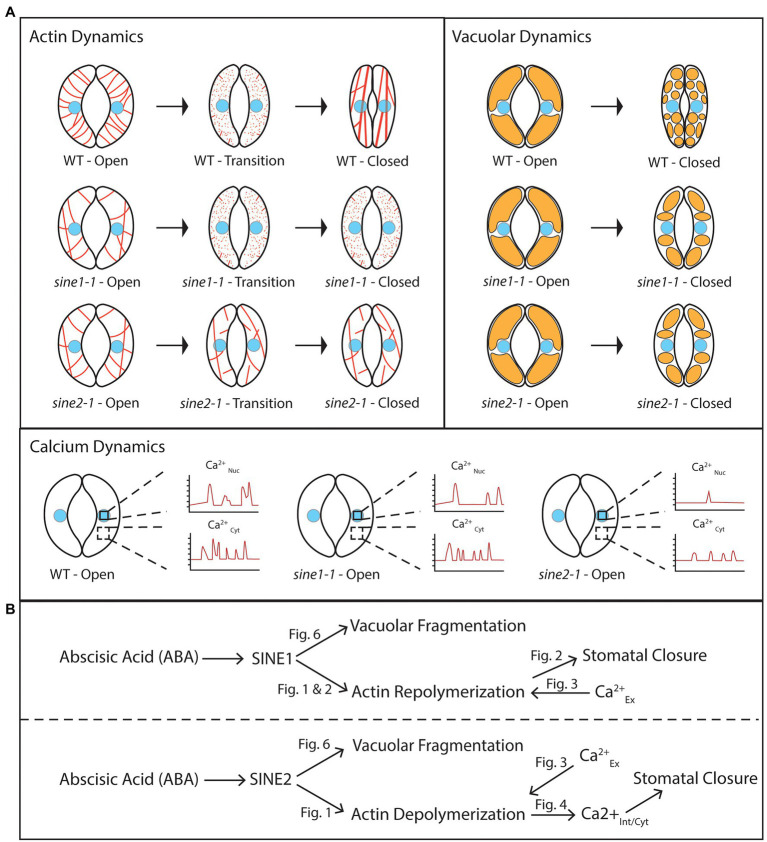
SINE1 and SINE2 have differential roles in ABA-induced guard cell closure. **(A)** Cartoon depicting subcellular dynamics during stomatal closure in WT and *sine* mutants. The cell nucleus is colored light blue. Actin is depicted in red, and vacuoles in orange. Ca_Nuc_, nuclear calcium fluctuations; Ca_Cyt_, cytoplasmic calcium fluctuations. **(B)** A working model proposing roles for SINE1 and SINE2 in stomatal closure based on the data shown here. “Fig” labels indicate figures showing the supportive experiment(s). Calcium^ex^, externally applied calcium; calcium^int/nuc^, internally measured nuclear calcium; calcium^int/cyt^, internally measured cytoplasmic calcium. For details, see text.

Conversely, loss of SINE2 leads to a mixed F-actin pattern throughout ABA-induced stomatal closure, with little to no depolymerization and little or no reorganization. JK did not rescue the closing defect of *sine2-1*, whether in the presence or absence of ABA (Biel et al., 2020a). Thus, loss of SINE2 appears to impair the early depolymerization stage of F-actin remodeling during guard cell closure, such that actin remains in an (inhibitory) filamentous state that is not altered by addition of JK (Figure 8A, left panel).

Similar effects on actin reorganization have been observed in mutants of subunits of the ARP2/3 complex, an important nucleation- and branching-inducing complex for F-actin and its upstream regulator the SCAR/WAVE complex. In an *arpc2* mutant, a more radial array of F-actin is maintained after ABA treatment, concomitant with delayed stomatal closure. Cytochalasin D restores this defect, suggesting that depolymerization of F-actin is required for closure. Similarly, mutating the PIR1 gene, which encodes a SCAR/WAVE subunit, inhibits dark-induced stomatal closure, and this can be restored by Cytochalasin D. These findings resemble our data on *sine2-1* (Jiang et al., 2012; Isner et al., 2017). Interestingly, *pir1* mutants have no defects in ABA-induced stomatal closure, giving room to the speculation that alternative ARP2/3 activators might exist in this signaling pathway and that SINE2 might be a candidate for such an activator.

SCAB1 is a plant-specific actin-binding protein that can stabilize and bundle actin filaments. Overexpression or loss of SCAB1 results in impaired ABA-induced stomatal closure associated with increased and decreased F-actin stability, respectively (Zhao et al., 2011). Interestingly, mutation of *SCAB1* also affects vacuolar remodeling during stomatal closure. SCAB1 activity is regulated by the important signaling molecule phosphatidylinositol 3-phosphate (PI3P) and the expression of PI3P biosynthesis genes is induced by ABA treatment (Yang et al., 2021).

Similarly, *sine1-1* mutants displayed decreased F-actin stability and *sine2-1* mutants had increased F-actin stability. While the potential regulation of SINE1 and SINE2 activity during stomatal remodeling is currently not known, the data together support a model where F-actin must be actively reorganized in a spatio-temporal manner in order to induce stomatal closure. Zhao et al. (2016) depict a model in which the actin depolymerizing factor ADF4 has increased activity during ABA-induced stomatal closure to depolymerize F-actin and is subsequently inhibited. Our data are consistent with a similar, currently unknown regulation of SINE2 activity.

We have shown previously that there is also an interaction between SINE1 and SINE2 and microtubules (MTs) in guard cells (Biel et al., 2020b). Depolymerizing MTs using oryzalin can restore ABA-induced stomatal closure deficits in *sine1-1* and *sine2-1* mutants. Loss of SINE1 or SINE2 results in loss of radially organized MT patterning in open guard cells, aberrant MT organization during stomatal closure, and an overall decrease in the number of MT filaments or bundles (Biel et al., 2020b). The role of microtubules in guard cells is a long-debated topic with evidence both for and against their involvement and is still poorly understood (reviewed in Li et al., 2022). However, because of recent evidence in actin-microtubule cross talk and connectivity, including work from plants (Schneider and Persson, 2015), it will be interesting to investigate in future work whether SINE1 and SINE2 could also play a role at such an intersection.

We have shown previously that addition of exogenous Ca^2+^ partially rescues the ABA-induced stomatal closure defect observed in *sine1-1* and *sine2-1* (Biel et al., 2020a). Additionally, utilizing the Ca^2+^ dye Fura-2, we showed that ABA-induced cytoplasmic Ca^2+^ may be perturbed in *sine1-1* and *sine2-1* (Biel et al., 2020a), thus suggesting that SINE1 and SINE2 act upstream of a Ca^2+^-dependent step in the ABA pathway. However, when using more robust genetically encoded Ca^2+^ sensors, we demonstrate here that ABA-induced changes in cytoplasmic Ca^2+^ oscillations are impaired in *sine2-1* but not *sine1-1*. Treatment with exogenous Ca^2+^ can rescue this defect in *sine2-1*. Treatment with LatB also rescues this phenotype, suggesting that actin depolymerization facilitated by SINE2 is required for cytoplasmic Ca^2+^ oscillations.

Previous studies have shown connections between actin reorganization and increases in Ca^2+^ during ABA-induced stomatal closure. In *Vicia faba*, guard cell stretch-activated and voltage-gated Ca^2+^ channels are activated by F-actin disruption and blocked by actin stabilization (Zhang et al., 2007; Zhang and Fan, 2009). In *Commelina communis*, addition of exogenous Ca^2+^ induces actin reorganization similar to that of ABA, and chelation of Ca^2+^ abolishes this reorganization in response to ABA (Hwang and Lee, 2001). Taken together with our data, this suggests that the absence of actin depolymerization in *sine2-1* might lead to perturbation of cytoplasmic Ca^2+^ spiking due to reduced plasma membrane Ca^2+^ channel activation.

In addition to the cytoplasmic Ca^2+^ defect, we show that nuclear Ca^2+^ fluctuations are also disrupted in *sine2-1*. While this is the first evidence of a potential role for changes in nuclear Ca^2+^ in guard cell abiotic stress response, previous studies have shown nuclear Ca^2+^ responses to abiotic and biotic stress in other cell types. An example is root symbiosis, where Ca^2+^ oscillations in and around the nucleus are required for host plant transcriptional response after perception of nodulation (Nod) or mycorrhizal (Myc) factors (Charpentier, 2018). Previous studies have also demonstrated that cytoplasmic and nuclear Ca^2+^ responses can occur independent from each other but coordinate to respond to stimuli (Charpentier et al., 2016; Huang et al., 2017; Kelner et al., 2018; Leitão et al., 2019). However, the relationship between cytoplasmic and nuclear Ca^2+^ oscillations in guard cells remains unclear. Dual-expressing Ca^2+^ sensors can now be used to further investigate and compare frequency and fluctuation characteristics between the guard cell nucleus and cytoplasm (Leitão et al., 2019). It will be interesting to investigate if nuclear and cytoplasmic Ca^2+^ coordinate in guard cells in a similar manner.

The ABA-induced vacuolar fragmentation observed during stomatal closure is reduced in *sine1-1* and *sine2-1*. Treatment with LatB partially overrides this phenotype, suggesting that some aspect of the actin patterns seen with loss of SINE1 or SINE2 is inhibitory to vacuolar fragmentation and that vacuolar fragmentation is downstream of SINE1 and SINE2-mediated changes in actin organization (Figure 8B). Previous work has shown that efficient actin organization is required for mediating changes in vacuolar morphology, and for the regulation of stomatal dynamics. Li et al. (2013) showed that the disruption of F-actin dynamics, using cytochalasin D (which inhibits actin polymerization) and phalloidin (which inhibits actin depolymerization), inhibited vacuolar fusion during stomatal opening. Vacuolar fusion was also impaired in the actin polymerization mutants *arp2* and *arp3*, in which vacuoles stay fragmented after delayed stomatal opening and actin organization is impaired (Li et al., 2013). Zheng et al. (2019) showed that it takes longer to reshape the vacuoles of guard cells in an actin severing mutant, *ap3m*, which also exhibited delayed stomatal closure.

Interestingly, exogenous Ca^2+^ resulted in stomatal closure in the absence of vacuolar fragmentation, regardless of the status of actin organization (Figure 7). This suggests that the cytoplasmic Ca^2+^ increase might be downstream of—or parallel to—vacuolar fragmentation, and that vacuolar fragmentation was not required for stomatal closing. Given that exogenous Ca^2+^ partially restored the actin depolymerization defect of *sine2-1*, that LatB treatment partially restored vacuolar fragmentation in *sine2-1*, but that exogenous Ca^2+^ did not lead to vacuolar fragmentation, suggests that vacuolar fragmentation is most likely not part of the pathway ABA ➔ SINE2 ➔ actin depolymerization ➔ internal Ca^2+^ oscillations ➔ stomatal closure (Figure 8B). It is currently unclear from our model why the vacuolar fragmentation defect in *sine1-1* can be partially rescued by LatB. Given that *sine1-1* promotes more depolymerized actin, the *sine1-1* mutant and LatB treatment should act in a similar fashion. However, this model might be oversimplified, if—for example—the *sine1-1* defect leads to the accumulation of short F-actin fragments not resolved by our imaging technique, which are inhibitory to vacuolar fragmentation, and which are depolymerized by LatB.

Taken together, we provide evidence for where in the pathway of ABA-induced stomatal closure the two paralogous KASH proteins SINE1 and SINE2 play a role, as related to F-actin re-organization, cytoplasmic and nuclear Ca^2+^ fluctuations, and vacuolar remodeling. Future work should address what the molecular activities of SINE1 and SINE2 are, how the differences between SINE1 and SINE2 domain function and expression patterns relate to their separable roles, whether nuclear Ca^2+^ fluctuations play a role in guard cell signaling, and what the molecular connection is between ABA signaling and the nuclear periphery.

## Data Availability Statement

The raw data supporting the conclusions of this article will be made available by the authors, without undue reservation.

## Author Contributions

AB, MM, and IM designed the experiments. AB and MM performed and analyzed the experiments. AB, MM, NRG, and IM wrote and edited the manuscript. IM provided oversight and funding for the study. All authors contributed to the article and approved the submitted version.

## Funding

This work was funded by National Science Foundation grants to IM (NSF-1613501 and NSF-2023348).

## Conflict of Interest

The authors declare that the research was conducted in the absence of any commercial or financial relationships that could be construed as a potential conflict of interest.

## Publisher’s Note

All claims expressed in this article are solely those of the authors and do not necessarily represent those of their affiliated organizations, or those of the publisher, the editors and the reviewers. Any product that may be evaluated in this article, or claim that may be made by its manufacturer, is not guaranteed or endorsed by the publisher.
