# Supplementary material for: Distinct Roles for KASH Proteins SINE1 and SINE2 in Guard Cell Actin Reorganization, Calcium Oscillations, and Vacuolar Remodeling
Source: Front Plant Sci. 2022 May 6;13:784342. doi: 10.3389/fpls.2022.784342 (PMC9120628; doi:10.3389/fpls.2022.784342)
Supplement: Supplementary file 1 [file Data_Sheet_1.pdf]

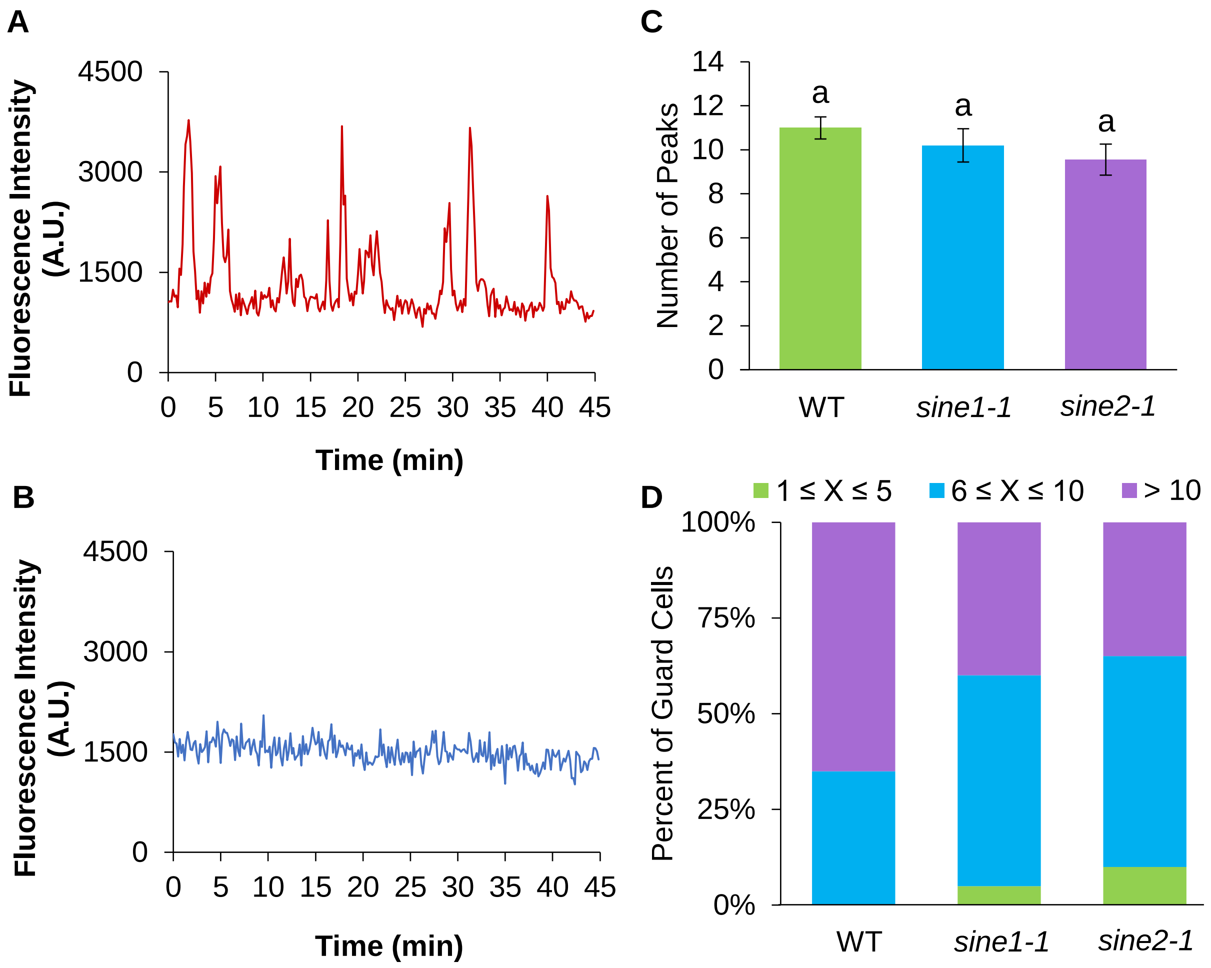

Supplementary Figure 1: Nuclear calcium fluctuations observed in opening buffer.

(A) Wild-type nuclear calcium fluctuations observed in full opening buffer presented as fluorescence intensity over time

(B) Wild-type nuclear calcium fluctuations observed in modified opening buffer, presented as fluorescence intensity over time

(C) Number of nuclear peaks observed after addition of full opening buffer in WT, *sine1-1*, and *sine2-1*

(D) Average number of nuclear peaks observed after addition of full opening buffer were grouped into several categories for WT, *sine1-1*, and *sine2-1*. Statistical significance was calculated using one-way ANOVA followed by a post hoc Tukey HSD test. Lowercase letters denote groups that are statistically different ($p<0.01$). All data are mean values ± SE from at least three independent experiments, with N = 20 guard cells.

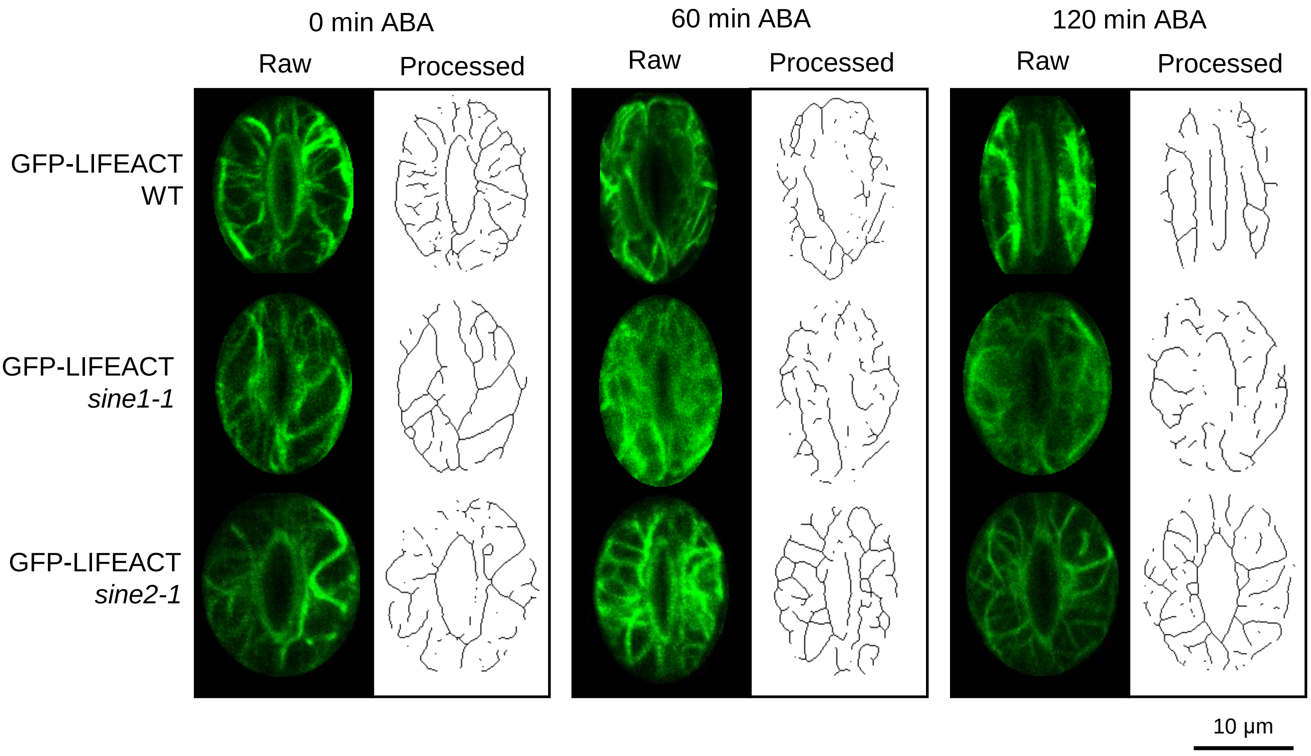

Supplementary Figure 2: Representative images demonstrating the skeletonization process of GFP-LIFEACT signal. “Raw” designates original images collected through confocal microscopy, while “Processed” images are the skeletonized image generated in ImageJ (see Materials and Methods).

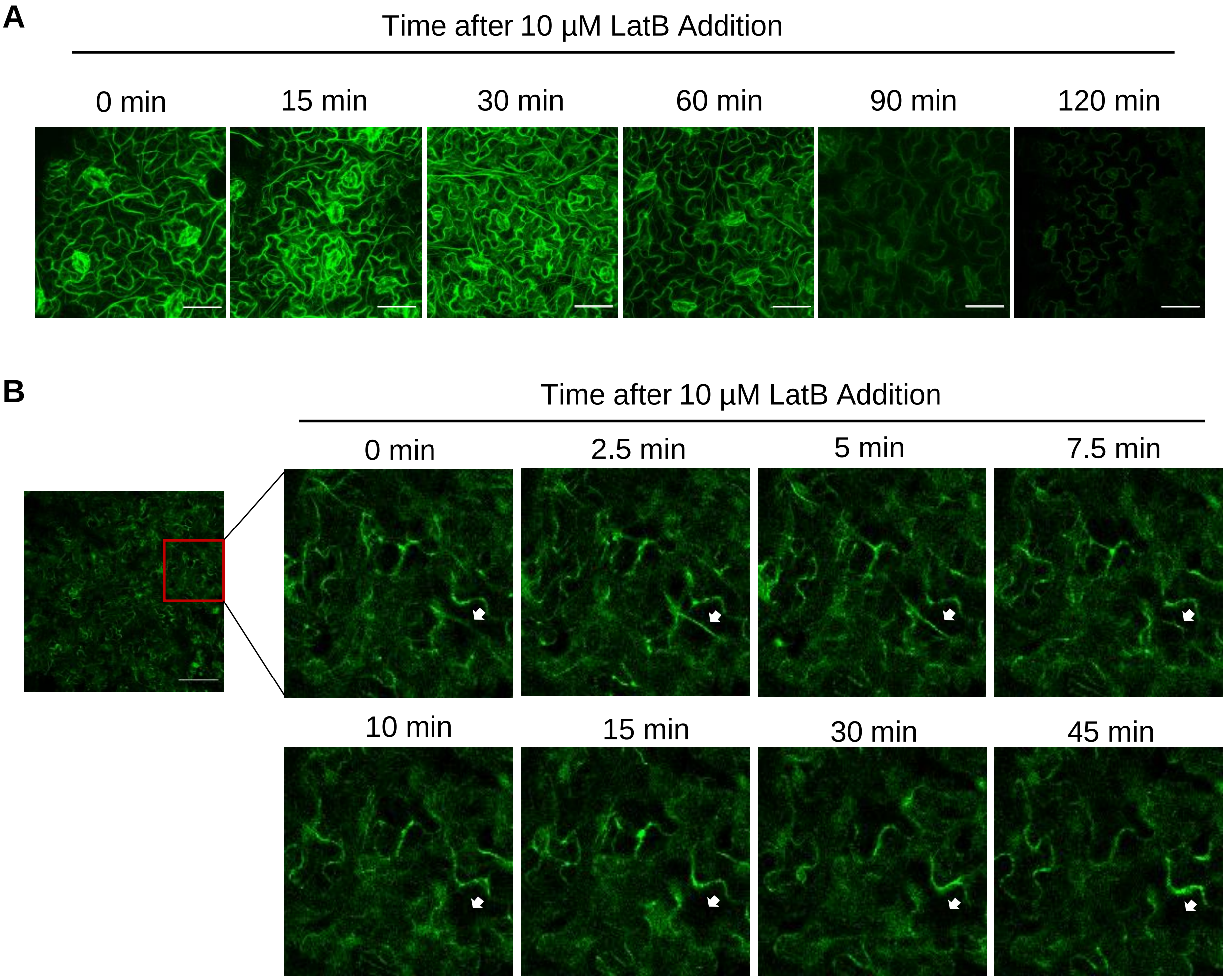

Supplementary Figure 3: Latrunculin B (LatB) diffuses F-actin signal in GFP-LIFEACT transgenic lines.

(A) Images of WT rosette leaves expressing GFP-LIFEACT at 0, 15, 30, 60, 90, and 120 min after 10 µM LatB addition. Scale bar = 20 µm

(B) Images of WT epidermal peel expressing GFP-LIFEACT at 0, 2.5, 5, 7.5, 10, 15, 30, and 45 min after 10 µM LatB addition. White arrow points to an actin filament of interest. Scale bar = 50 µm

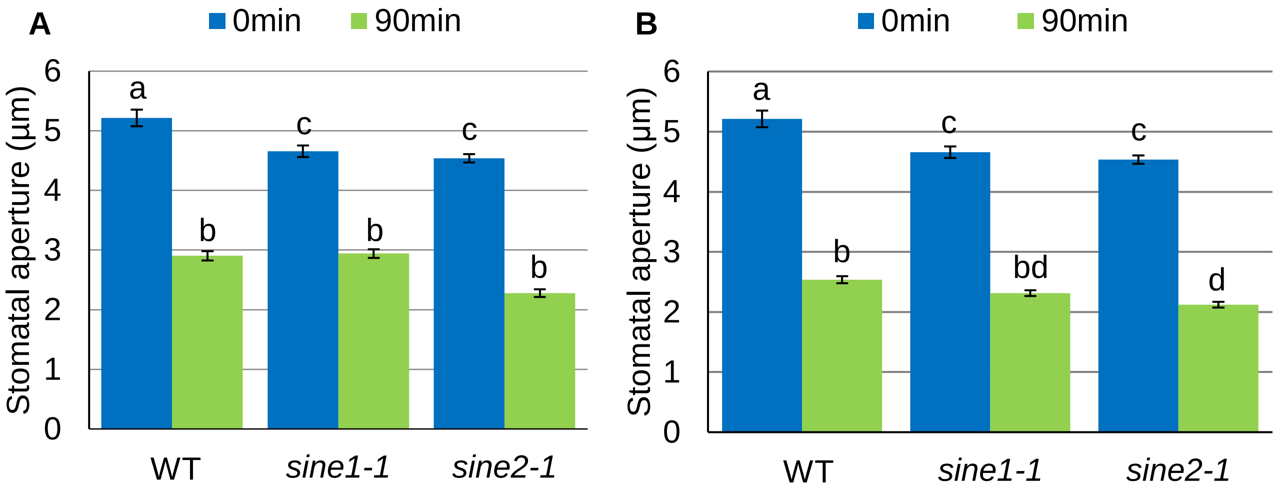

Supplementary Figure 4: Calcium induces stomatal closure with or without actin depolymerization. Stomatal aperture measurements are shown for 0 min and 90 min before and after induced closure with:

(A) 10 mM Ca^{2+}

(B) 10 mM Ca^{2+} + 10 µM LatB

Ca^{2+}-induced stomatal closure assays were used here as described in section “Materials and Methods”, utilizing WT, *sine1-1*, and *sine2-1*. Statistical significance was calculated using one-way ANOVA followed by a post hoc Tukey HSD test. Lowercase letters denote groups that are statistically different ($p<0.01$). All data are mean values ± SE from three independent experiments, with N ≥ 60 stomata.

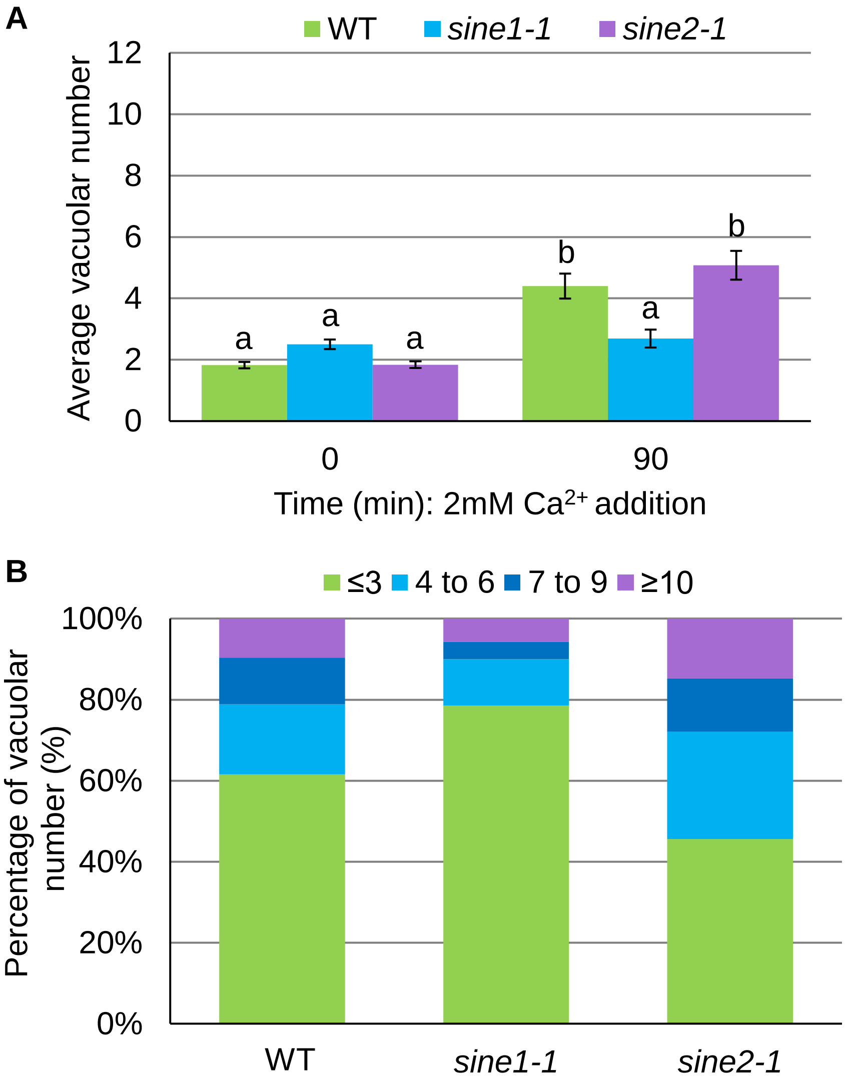

Supplementary Figure 5: 2mM Ca^{2+} induces minimal fragmentation in guard cells.

(A) Average vacuolar number seen in guard cells before and after 2mM Ca^{2+} treatment

(B) The number of distinct vacuolar structures observed in guard cells were counted and binned into numerical categories, shown here as percentages, after 90 min of treatment 2mM Ca^{2+} treatment.

Statistical significance was calculated using one-way ANOVA followed by a post hoc Tukey HSD test. Lowercase letters denote groups that are statistically different ($p<0.01$). All data are mean values ± SE from three independent experiments, with N ≥ 68 guard cells.

Primer Name	Sequence (5'– 3')	Construct
GC1ProSacIF	**cacc**gagctcatggttgcaacagagaggatg	GC1 promoter
GC1ProSpeIR	actagtatttcttgagtagtgattttgaagtag	GC1 promoter
NES-YC3.6F	**cacc**atgctgcagaacgagcttgc	NES-YC3.6 calcium sensor
NES-YC3.6R	gctctgcagccaactagtttactcgatgtt	NES-YC3.6 calcium sensor
NLS-R-GECO1F	**cacc**atgccaaaaaagaagcggaaggtcgatcc	NLS-R-GECO1 calcium sensor
NLS-R-GECO1R	ctacttcgctgtcatcatttgtaca	NLS-R-GECO1 calcium sensor

Supplementary Table 1: Primers used for cloning. CACC sites for directional TOPO cloning are indicated in bold. Specific recognition sites for *SacI* and *SpeI* are underlined.

90 min Closure	WT	*sine1-1*	*sine2-1*
**ABA vs ABA+LatB**	P=0.87	P<0.05	P<0.001
**ABA vs 2mM Ca^{2+}**	P<0.001	P<0.001	P<0.01
**2mM Ca^{2+} vs. 10mM Ca^{2+}**	P<0.001	P=0.39	P<0.001
**10mM Ca^{2+} vs. LatB+Ca^{2+}**	P=0.16	P=0.21	P=0.26

Supplementary Table 2: Extended analysis of statistical significance for the data shown in Figures 6 and 7, comparing average number of vacuoles. Comparisons are performed between 90 min time points of the following treatment groups, for individual lines: ABA (Fig. 6), ABA+LatB (Fig. 6), 2mM Ca2+ (Suppl Fig. 5), 10mM Ca2+ (Fig. 7), or Ca^{2+}+LatB (Fig. 7). Numbers shown are P-values from Student's t-test.
